# Association between spicy food consumption and the risk of non-alcoholic fatty liver disease/metabolic dysfunction-associated steatotic liver disease and liver fibrosis

**DOI:** 10.3389/fnut.2025.1729349

**Published:** 2025-11-26

**Authors:** Na Zhao, Huimin Liu, Yan Wang, Yun He, Ning Zhang, Yuan Li

**Affiliations:** Department of Gastroenterology and Hepatology, The Second Hospital of Hebei Medical University, Shijiazhuang, China

**Keywords:** spicy food, non-alcoholic fatty liver disease, metabolic dysfunction-associated steatotic liver disease, advanced liver fibrosis, cohort study

## Abstract

**Introduction:**

Evidence suggested that capsaicin may protect against steatotic liver disease (SLD), but these findings lack validation in population-based studies. This research aimed to explore the association between spicy food consumption and the risk of non-alcoholic fatty liver disease (NAFLD)/metabolic dysfunction-associated steatotic liver disease (MASLD) and liver fibrosis.

**Methods:**

A total of 23,666 participants aged 25 to 60, free from NAFLD, MASLD, and liver fibrosis, were recruited from a multi-center physical examination database in Shijiazhuang, Hebei Province, China, between 2011 and 2024. Cox proportional hazards regression model assessed the association between spicy food consumption and incident NAFLD/MASLD and advanced liver fibrosis. Restricted cubic spline (RCS) functions estimated the dose–response relationship. Subgroup and sensitive analyses evaluated heterogeneity based on various characteristics, while sensitivity analyses tested the robustness of results.

**Results:**

There were 42.2% of participants who reported consuming spicy food at least once per week. In this cohort study, a total of 7,965 patients with NAFLD and 7,311 patients with MASLD were identified after a median follow-up period of 12.6 years. Those who consumed spicy food more than once a week had a significantly lower risk of NAFLD/MASLD compared to non-consumers, indicating a dose–response relationship. However, this association was not observed in advanced liver fibrosis.

**Conclusion:**

Weekly spicy food consumption was inversely associated with risk of incident NAFLD/MASLD, but not advanced liver fibrosis.

## Introduction

The burden of NAFLD remained significant both globally and within China, characterized by high prevalence and incidence rates. NAFLD has demonstrated a global prevalence exceeding 25%, with an average annual percent change (AAPC) of 0.72% (95% Confidential Interval [CI] = 0.67–0.77%) observed from 1990 to 2021 (1, 2). In 2021, China reported approximately 287.5 million prevalent cases (95% Uncertainty Interval [UI] = 261.6–314.1 million), alongside an estimated 8.8 million incident cases (95% UI = 8.6–8.9 million), resulting in approximately 6,300 deaths (95% UI = 4,000–9,200) and around 158,000 disability-adjusted life years (DALYs) lost (95% UI = 102,100–231,400) (3). However, according to the Delphi consensus process conducted in 2023, it has been proposed to adopt the term “steatotic liver disease” as a replacement for “fatty liver disease.” Furthermore, it is recommended that the terminology “metabolic dysfunction-associated steatotic liver disease” be utilized instead of “NAFLD” (4). A similar trend was observed regarding the global prevalence and incidence of MASLD; notably, the largest increases in age-standardized point prevalence estimates from 2010 to 2021 were recorded in China at a rate of 16.9% (95% UI = 14.7–18.9%). Furthermore, both the incidence and prevalence of MASLD are rapidly escalating during this period (5).

NAFLD/MASLD represented a disease spectrum ranging from steatosis, with or without mild inflammation (non-alcoholic fatty liver), to non-alcoholic steatohepatitis (NASH). NASH was characterized by necroinflammation and more rapid fibrosis progression than non-alcoholic fatty liver, making it a leading cause of cirrhosis and hepatocellular carcinoma (6, 7). Although cardiovascular disease and extrahepatic malignancies were the leading causes of mortality in individuals with NAFLD, advanced liver fibrosis served as a critical prognostic indicator for both liver-related outcomes and overall survival (8–10). This condition can be evaluated through various combinations of non-invasive tests. A minority of patients may experience inflammation, which increases the risk of progressive fibrosis that could lead to cirrhosis (11). The progression to cirrhosis occurred in approximately 3–5% of affected individuals, often taking more than two decades (12). Although several therapeutic agents were currently in advanced stages of development, there was presently no approved pharmacological treatment for NAFLD. Therefore, adopting a healthy lifestyle and achieving weight reduction remained essential strategies for both the prevention and management of this condition (13, 14).

Spicy foods, including chili peppers, sweet peppers, hot red chili peppers, and fermented red peppers—as well as any food containing these ingredients—have been proved to be related to many outcomes in previous studies. These included psychological health (15), fractures (16), hyperuricemia (17), obesity (18, 19), type 2 diabetes mellitus (DM) (20), irritable bowel syndrome (21), cardiovascular diseases (22–24), cancers (25), overall mortality, and cause-specific mortality (26, 27). Capsinoids (i.e., capsiate, dihydrocapsiate, nordihydrocapsiate), capsaicinoids (i.e., capsaicin, dihydrocapsaicin, nordihydrocapsaicin, homodihydrocapsaicin, homocapsaicin), and capsiconinoids (i.e., capsiconiate, dihydrocapsiconiate), which were primarily found in chili peppers, have been demonstrated to be closely associated with the regulation of lipid deposition. Additionally, they improved cholesterol metabolism and insulin resistance while decreasing oxidative stress and reducing apoptotic cell death both *in vitro* and in animal studies (28–31). Moreover, numerous mechanistic studies suggested that the protective effects of capsaicin against NAFLD and SLD were attributed to its anti-steatotic, antioxidant, anti-inflammatory, and anti-fibrotic properties. Furthermore, capsaicin has shown promising potential in inhibiting metabolic syndrome and gut dysbiosis while promoting bile acid production; these actions contributed significantly to its role in combating NAFLD (32). However, it was important to note that these findings have not yet been validated in population-based studies, particularly within large cohort populations.

Therefore, we aimed to investigated the relationship between spicy food consumption and the risk of NAFLD/MASLD as well as advanced liver fibrosis using a large Chinese population cohort.

## Methods

### Study design and population

This study focused on longitudinal data and large population cohorts. The study population was gathered from a multi-center physical examination database located in Shijiazhuang, Hebei Province, China, during the period from October 2011 to October 2024. Follow-up continued until the date of the endpoint of observation on October 31, 2024. Each participant’s follow-up began at enrollment and concluded upon an occurrence of NAFLD/MASLD, loss to follow-up, or at the endpoint of observation in October 31, 2024. Participants who engaged in at least twelve sessions during the 14-year follow-up period were eligible for inclusion in the study; notably, 46% of the population participated in all fourteen rounds of surveys. All participants were required to complete all physical examinations before leaving each session. Further diagnosis and treatment would be necessary if any medical conditions were identified during these examinations.

The data collected through lifestyle survey questionnaires (e.g., sociodemographic characteristics, dietary preferences, family and personal medical history), physical measurements (e.g., height, body weight, and blood pressure), clinical laboratory tests (e.g., blood, urine, and feces), and abdominal ultrasound examinations (e.g., liver, gallbladder, pancreas, spleen, and kidney). Standardized questionnaire surveys were administered prior to the participants’ physical examinations. During this period, a total of 23,666 Chinese individuals aged 25–60 years who completed both the questionnaire survey and physical examinations were recruited for this study. All participants were free from NAFLD/MASLD and advanced liver fibrosis as identified by abdominal ultrasound examination and clinical laboratory tests at baseline. Their institutions or companies organized the physical examinations, respectively, per year.

This research was granted ethical approval by the Institutional Review Board (IRB) of the Second Hospital of Hebei Medical University. Informed consent was obtained from all participants involved in the study. The procedures adhered to the Declaration of Helsinki and other applicable regulations.

### Definitions of spicy food consumption and chili level preference

We assessed the frequency of spicy food consumption over the course of 1 week utilizing a structured questionnaire. Two questions were employed to assess spicy food intake. The first question was: “How often do you consume spicy foods in past week over the past months?” Participants were instructed to respond by selecting one of the following options: never, less than 1 day per week, 1–2 days per week, 3–5 days per week, or 6–7 days per week. Spicy food intake was defined as follows: direct consumption of fresh chili peppers; fresh/fermented/dried chili peppers; sweet peppers; chili oil; chili sauce/paste; curry; or other hot red chili peppers used in cooking. A subsequent question was directed to participants who reported consuming spicy foods at least once per week: “What is your preferred level of spiciness?” Participants were presented with three options for spiciness levels: heavy, moderate, and mild.

### Definitions of other variables

In this study, data were collected from three dimensions to explore potential associations between spicy food consumption and NAFLD/MASLD or liver fibrosis. Sociodemographic characteristics included age, gender, smoking and drinking status, educational condition, work intensity, household income, and marital status. The smoking status of participants was assessed through questions to determine if they currently smoke. Excessive alcohol consumption was assessed via a questionnaire, defined as exceeding 30 grams per day for male participants and exceeding 20 grams per day for female participants (4). The educational level was categorized as either below a university degree or at the university degree level and above. The intensity of work was self-reported by participants, categorized as either light or heavy. A high household income was defined as an annual household income exceeding 150 thousand yuan. Marital status was categorized into two groups: currently married or not. Dietary preferences across all survey waves included weekly consumption of vegetables, fruits, meat, and eggs; responses were categorized into two groups: frequent and seldom.

Anthropometric variables, including waist circumference (WC), height, and body weight, were measured following a standardized protocol. Body mass index (BMI) was calculated as body weight in kilograms divided by height in meters squared. Blood pressure readings were obtained as the average of three distinct measurements taken from the upper right arm at the brachial artery, utilizing an automatic device following a 5-min rest period in a seated position. In this study, we collected blood samples for various indices related to blood glucose and lipid metabolism, including total cholesterol (CHOL), triglycerides (TG), high-density lipoprotein (HDL), low-density lipoprotein (LDL), platelet count (PLT), albumin (Alb), alanine aminotransferase (ALT), aspartate aminotransferase (AST), total bilirubin (TBil), glycosylated hemoglobin (HbA1c), fasting blood glucose (FBG), and direct bilirubin (DBil).

Family medical history was systematically gathered, with a particular emphasis on three prevalent health conditions: diabetes, hypertension, hepatitis, and cancer. Data concerning the age of onset, as well as the duration for which family members had been diagnosed and treated, were meticulously documented.

### Definitions of NAFLD/MASLD and advanced liver fibrosis

Abdominal ultrasonography was utilized to investigate SLD. A skilled technician conducted the liver ultrasound, and all images were prospectively assessed by experienced hepatologists who were blinded to the clinical data. SLD was defined by positive ultrasound findings, characterized by two or more abnormal results in liver ultrasonography. Specifically, these criteria included: (1) diffusely increased echogenicity of the liver; (2) heightened echogenicity of the liver compared to that of the kidney or spleen; and (3) vascular blurring accompanied by gradual attenuation of the ultrasound signal. The ultrasonographic assessment of the SLD was classified as normal, mild, moderate, or severe hepatic steatosis.

NAFLD was defined as the presence of steatosis in individuals without excessive alcohol consumption or evidence of active hepatitis viral infection (33). Participants who tested positive for hepatitis B surface antigen or exhibited positive antibodies for hepatitis C virus were excluded from this study based on their blood laboratory test results.

MASLD referred to steatosis that is associated with the presence of at least one indicator of cardio-metabolic dysregulation. This condition was characterized by the occurrence of at least one of the following abnormalities related to cardio-metabolic risk: (1) a BMI ≥ 24 kg/m^2^ or WC ≥ 94 cm for male participants or ≥80 cm for female participants; (2) systolic blood pressure (SBP) ≥ 130 mmHg, diastolic blood pressure (DBP) ≥ 85 mmHg, or receipt of specific anti-hypertensive medication; (3) plasma TG ≥ 1.70 mmol/L, or treatment with lipid-lowering medications; (4) plasma HDL < 1.0 mmol/L for male participants and <1.3 mmol/L for female participants, or treatment with lipid-lowering medications; and (5) pre-diabetes indicated by FBG levels between 5.6–6.9 mmol/L, glycated hemoglobin levels ≥5.7%, diagnosis of type 2 DM, or treatment with specific anti-diabetic medications (34).

The NAFLD Fibrosis Score (NFS) and the Fibrosis-4 index (FIB-4) were utilized to assess advanced liver fibrosis, which was widely employed in clinical practice for non-invasive evaluation of the risk of advanced liver fibrosis. Specifically, NFS was calculated using the following formula: NFS = −1.675 + 0.037 × age (years) + 0.094 × BMI (kg/m^2^) + 1.13 × diabetes (yes = 1, no = 0) + 0.99 × AST (U/L)/ALT (U/L) − 0.013 × PLT (×10^9^/L) − 0.66 × Alb (g/dL). A NFS value greater than −1.455 indicated advanced fibrosis (35). FIB-4 was developed using the formula: FIB-4 = (age [years] × AST [U/L])/((PLT [×10^9^/L]) × (ALT [U/L]) ^ (1/2)). A FIB-4 score exceeding 1.3 suggested advanced fibrosis (36).

### Statistical analysis

The multiple imputation approach was employed to handle missing values. We utilized a binary logistic regression model to investigate the mechanism underlying the missing data and subsequently generated imputed datasets in accordance with this mechanism. With less than 5% of the data missing, we handled them using the Multivariate Imputation by Chained Equations (MICE) approach. The imputation model included all variables intended for subsequent analyses. Continuous variables were imputed using predictive mean matching, and categorical variables were imputed using logistic regression. We created 20 imputed datasets, running 10 iterations per dataset to ensure model stability and convergence. Subsequently, the results from analyses on these 20 datasets were pooled following Rubin’s rules (37–39). All imputations were performed using the mice package in R software.

The distribution of baseline variables was presented according to the reported frequency of consumption of spicy foods. Continuous variables were expressed as means with 95% confidence intervals (CIs), while categorical variables were displayed as frequencies and percentages. The relationship between spicy food consumption/chili level preference and the risk of NAFLD/MASLD, as well as advanced liver fibrosis, was assessed using Cox proportional hazards regression models across different variables. These adjusted models examined the association between the frequency of spicy food consumption and the risks associated with NAFLD/MASLD and liver fibrosis. Model I provided crude hazard ratios (HRs) along with 95% CIs; Model II further adjusted for demographic factors; Model III included additional adjustments for dietary preferences, and family medical history. Given that markers of obesity, blood pressure, blood glucose, or liver function may serve as intermediate factors in the potential causal pathway linking spicy food consumption to NAFLD/MASLD and advanced liver fibrosis, we excluded them from the multivariate analyses. In all analyses, participants who never consumed spicy foods were the reference category. For further analyses on chili level preference, those who never consumed spicy foods and those who consumed them less than once a week served as the reference category. HRs along with 95% CIs were utilized to compare risks among various groups. To evaluate trends in HRs across increasing categories of spicy food intake, we employed a Mantel–Haenszel extension chi-square test.

Cox proportional hazards regression models with adjusted RCS were used to explore the nonlinear relationship between spicy food consumption frequency and the risk of incident NAFLD/MASLD and liver fibrosis. Participants who never consumed spicy foods served as the reference category for all analyses of nonlinear associations. Nonlinear curve fitting was optimized by including three knots in the models, reducing accuracy loss from over-fitting (40).

Additionally, we conducted subgroup analyses by categorizing several demographic covariates for further study. These analyses included subgroups based on age group, sex, smoking status, educational level, work intensity, household income, and marital status. Furthermore, sensitivity analyses were performed to ensure the robustness of our findings across three distinct aspects. First, we compared results before and after including participants with missing data. Second, we assessed results prior to and following multiple imputation procedures. Lastly, we excluded participants with a reported family medical history to evaluate the stability of our conclusions.

## Results

### Multiple imputation of missing data

As presented in Supplementary Table 1, we observed that the proportion of missing data across all variables was less than 5%. Furthermore, our logistic regression analysis investigating the relationship between all variables with missing data and NAFLD/MASLD as well as advanced liver fibrosis revealed that all *p*-values were non-significant, as shown in Supplementary Table 2. These findings indicate that the missing data for the independent variables are independent of the dependent variables in this study. Consequently, we infer that the missing data can be classified as missing at random.

After imputing the missing data using multiple imputation techniques, we found that the distributions of the imputed values closely mirrored those of the observed values. As illustrated in Supplementary Figure 1, gray represented observed data while red depicts imputed data.

### Baseline characteristics of participants

Detailed baseline information on participants categorized by the frequency of weekly spicy food consumption in this study was shown in Table 1. A total of 23,666 participants aged 25–60 years, free of NAFLD/MASLD and advanced liver fibrosis, were enrolled between 2011 and 2024. The average age of the participants was 40.0 years (95% CI = 39.8–40.1) in 2011. Among the participants, 9,990 (42.2%) reported consuming spicy food at least once per week. Specifically, there were 5,930 participants who reported never consuming spicy food; 7,746 participants consumed spicy food less than once per week; 2,587 participants consumed spicy food one to two days per week; 2,431 participants consumed spicy food three to five days per week; and 4,972 participants consumed spicy food six to seven days per week. A total of 7,965 patients with NAFLD and 7,311 patients with MASLD were identified after a 12.6-year median follow-up in this cohort study. Among these individuals, there were 342 diagnosed with advanced liver fibrosis based on the Nonalcoholic Fatty Liver Disease Fibrosis Score (NFS), and an additional 380 diagnosed as having advanced liver fibrosis based on the FIB-4 index.

**Table 1:** Baseline characteristics of participants categorized by the frequency of weekly spicy food consumption.

Characteristics	Frequency of weekly spicy food consumption					All participants
	Never	< 1 day/week	1–2 days/week	3–5 days/week	6–7 days/week	
Number of participants	5,930	7,746	2,587	2,431	4,972	23,666
Demographic factors						
Age [years; mean (95% CI)]	39.8 (39.5–40.1)	40.2 (39.9–40.4)	40.3 (39.9–40.8)	40.3 (39.8–40.7)	39.6 (39.3–40.0)	40.0 (39.8–40.1)
Sex, Female, *n* (%)	2,953 (49.8%)	3,848 (49.7%)	1,288 (49.8%)	1,248 (51.3%)	2,456 (49.4%)	11,793 (49.8%)
Current smoker, *n* (%)	731 (12.3%)	905 (11.7%)	306 (11.8%)	308 (12.7%)	644 (13.0%)	2,894 (12.2%)
Excessive alcohol consumption, *n* (%)	522 (8.8%)	612 (7.9%)	215 (8.3%)	182 (7.5%)	388 (7.8%)	1919 (8.1%)
Advanced education, *n* (%)	1967 (33.2%)	2,544 (32.8%)	863 (33.4%)	822 (33.8%)	1,597 (32.1%)	7,793 (32.9%)
High work intensity, *n* (%)	588 (9.9%)	806 (10.4%)	238 (9.2%)	263 (10.8%)	522 (10.5%)	2,417 (10.2%)
High household income, *n* (%)	752 (12.7%)	988 (12.8%)	321 (12.4%)	325 (13.4%)	632 (12.7%)	3,018 (12.8%)
Currently married, *n* (%)	5,024 (84.7%)	6,545 (84.5%)	2,220 (85.8%)	2073 (85.3%)	4,223 (84.9%)	20,085 (84.9%)
Dietary preferences						
Vegetables, *n* (%)	5,810 (98.0%)	7,584 (97.9%)	2,536 (98.0%)	2,377 (97.8%)	4,849 (97.5%)	23,156 (97.8%)
Fruits, *n* (%)	2,804 (47.3%)	3,721 (48.0%)	1,180 (45.6%)	1,161 (47.8%)	2,387 (48.0%)	11,253 (47.5%)
Meat, *n* (%)	4,730 (79.8%)	6,155 (79.5%)	2080 (80.4%)	1964 (80.8%)	3,955 (79.5%)	18,884 (79.8%)
Egg, *n* (%)	5,071 (85.5%)	6,625 (85.5%)	2,212 (85.5%)	2091 (86.0%)	4,245 (85.4%)	20,244 (85.5%)
Physical measurements						
BMI [kg/m^2^; mean (95% CI)]	24.0 (23.9–24.1)	24.0 (23.9–24.1)	23.9 (23.8–24.0)	23.9 (23.8–24.1)	24.0 (23.9–24.1)	24.0 (23.9–24.0)
WC [cm; mean (95% CI)]	81.0 (80.8–81.2)	81.2 (81.0–81.3)	80.7 (80.5–81.0)	80.9 (80.7–81.2)	80.9 (80.7–81.1)	81.0 (80.9–81.1)
DBP [mmHg; mean (95% CI)]	79.7 (79.6–79.9)	79.9 (79.7–80.0)	79.9 (79.6–80.1)	80.0 (79.8–80.3)	80.0 (79.9–80.2)	79.9 (79.8–80.0)
SBP [mmHg; mean (95% CI)]	132.5 (132.2–132.7)	132.5 (132.2–132.7)	132.7 (132.3–133.1)	132.7 (132.3–133.1)	132.4 (132.1–132.7)	132.5 (132.4–132.6)
CHOL [mmol/L; mean (95% CI)]	4.51 (4.49–4.53)	4.49 (4.47–4.51)	4.50 (4.47–4.53)	4.50 (4.46–4.53)	4.51 (4.48–4.53)	4.50 (4.49–4.51)
TG [mmol/L; mean (95% CI)]	1.34 (1.33–1.35)	1.34 (1.33–1.35)	1.34 (1.32–1.36)	1.35 (1.33–1.37)	1.34 (1.32–1.35)	1.34 (1.33–1.35)
HDL [mmol/L; mean (95% CI)]	1.39 (1.39–1.40)	1.40 (1.39–1.40)	1.40 (1.39–1.40)	1.39 (1.39–1.40)	1.40 (1.39–1.40)	1.40 (1.39–1.40)
LDL [mmol/L; mean (95% CI)]	2.49 (2.48–2.51)	2.49 (2.48–2.50)	2.49 (2.47–2.51)	2.52 (2.49–2.54)	2.49 (2.48–2.51)	2.49 (2.49–2.50)
Alb [g/L; mean (95% CI)]	49.9 (49.9–50.0)	50.0 (49.9–50.0)	49.9 (49.8–50.0)	50.0 (49.9–50.1)	50.0 (49.9–50.0)	50.0 (49.9–50.0)
ALT [U/L; mean (95% CI)]	39.7 (39.4–40.0)	39.8 (39.5–40.0)	40.0 (39.5–40.4)	39.9 (39.4–40.4)	40.2 (39.9–40.5)	39.9 (39.7–40.0)
AST [U/L; mean (95% CI)]	29.9 (29.8–30.1)	29.9 (29.8–30.0)	29.7 (29.5–30.0)	30.0 (29.7–30.2)	30.0 (29.9–30.2)	29.9 (29.8–30.0)
PLT [×10^9/L; mean (95% CI)]	324.8 (324.4–325.2)	324.8 (324.5–325.1)	325.1 (324.5–325.6)	324.6 (324.0–325.2)	325.1 (324.7–325.5)	324.9 (324.7–325.1)
HbA1 [%; mean (95% CI)]	5.50 (5.46–5.55)	5.52 (5.49–5.56)	5.53 (5.46–5.59)	5.46 (5.39–5.52)	5.52 (5.47–5.57)	5.51 (5.49–5.53)
FBG [mmol/L; mean (95% CI)]	5.49 (5.47–5.52)	5.49 (5.47–5.52)	5.49 (5.44–5.53)	5.51 (5.46–5.55)	5.47 (5.44–5.50)	5.49 (5.48–5.50)
TBil [μmol/L; mean (95% CI)]	18.9 (18.7–19.0)	18.9 (18.8–19.0)	19.0 (18.8–19.2)	18.9 (18.7–19.1)	18.9 (18.8–19.1)	18.9 (18.8–19.0)
DBil [μmol/L; mean (95% CI)]	5.51 (5.46–5.55)	5.50 (5.46–5.54)	5.46 (5.40–5.53)	5.52 (5.45–5.59)	5.52 (5.47–5.56)	5.50 (5.48–5.52)
Chili level preference						
Mild, *n* (%)	–	–	2,393 (92.5%)	2052 (84.4%)	3,022 (60.7%)	7,467 (74.7%)
Moderate, *n* (%)	–	–	167 (6.4%)	314 (12.9%)	1,082 (21.8%)	1,563 (15.6%)
Heavy, *n* (%)	–	–	27 (1.0%)	65 (2.7%)	868 (17.5%)	960 (9.6%)
Family medical history						
Diabetes, *n* (%)	1,182 (19.9%)	1,548 (20.0%)	530 (20.5%)	475 (19.5%)	1,012 (20.4%)	4,747 (20.1%)
Hypertension, *n* (%)	1,272 (21.5%)	1,596 (20.6%)	525 (20.3%)	523 (21.5%)	1,048 (21.1%)	4,964 (21.0%)
Hepatitis, *n* (%)	456 (7.7%)	602 (7.8%)	218 (8.4%)	175 (7.2%)	420 (8.4%)	1871 (7.9%)
Cancer, *n* (%)	575 (9.7%)	787 (10.2%)	250 (9.7%)	204 (8.4%)	485 (9.8%)	2,301 (9.7%)
Health outcomes						
NAFLD, *n* (%)	2,322 (39.2%)	2,912 (37.6%)	847 (32.7%)	655 (26.9%)	1,229 (24.7%)	7,965 (33.7%)
MASLD, *n* (%)	2,236 (37.7%)	2,681 (34.6%)	812 (31.4%)	549 (22.6%)	1,033 (20.8%)	7,311 (30.9%)
Advanced liver fibrosis^a^	72 (1.21%)	107 (1.38%)	37 (1.43%)	41 (1.69%)	85 (1.70%)	342 (1.44%)
Advanced liver fibrosis^b^	113 (1.90%)	102 (1.32%)	36 (1.40%)	37 (1.52%)	92 (7.85%)	380 (1.60%)

BMI, body mass index; WC, waist circumstance; DBP, diastolic blood pressure; SBP, systolic blood pressure; CHOL, total cholesterol; TG, Triglyceride; HDL, high density lipoprotein; LDL, low density lipoprotein; Alb, albumin; ALT, alanine aminotransferase; AST, aspartate aminotransferase; PLT, platelet; HbA1, glycosylated hemoglobin; FBG, fast blood glucose; TBil, total bilirubin; DBil, direct bilirubin; NAFLD, non-alcoholic fatty liver disease; MASLD, metabolic dysfunction-associated steatotic liver disease.

^a^Advanced liver fibrosis was evaluated using the NAFLD Fibrosis Score (NFS), with advanced liver fibrosis defined as NFS > − 1.455.

^b^Advanced liver fibrosis was evaluated using the Fibrosis-4 index (FIB-4), with advanced liver fibrosis defined as FIB-4 > 1.3.

### Spicy food consumption and NAFLD/MASLD/advanced liver fibrosis

As shown in Table 2, after adjusting for covariates, participants consuming spicy food 1–2 days per week had an 18.9% lower risk of NAFLD (HR = 0.811, 95%CI = 0.750–0.878) and a 19.7% lower risk of MASLD (HR = 0.803, 95%CI = 0.741–0.871). Those consuming spicy food 3–5 days per week experienced a 33.2% lower risk of NAFLD (HR = 0.668, 95%CI = 0.612–0.729) and a 42.0% lower risk of MASLD (HR = 0.580, 95%CI = 0.528–0.637). Participants consuming spicy food 6–7 days per week had a 36.4% lower risk of NAFLD (HR = 0.636, 95%CI = 0.593–0.681) and a 44.6% lower risk of MASLD (HR = 0.554, 95%CI = 0.515–0.596), compared to those who never consumed spicy food. However, we did not observe a significant difference in the risk of advanced liver fibrosis between participants who consumed spicy foods and those who did not.

**Table 2:** Adjusted hazard ratios for the risk of NAFLD/MASLD and advanced liver fibrosis, stratified by the frequency of weekly consumption of spicy foods.

Health outcomes	Model I (HR [95% CI])^a^	Model II (HR [95% CI])^b^	Model III (HR [95% CI])^c^
NAFLD			
Never	Ref.	Ref.	Ref.
< 1 day/week	0.932 (0.882–0.985)	0.935 (0.885–0.988)	0.945 (0.894–1.008)
1–2 days/week	0.801 (0.740–0.868)	0.799 (0.738–0.866)	0.811 (0.750–0.878)
3–5 days/week	0.670 (0.614–0.730)	0.675 (0.619–0.735)	0.668 (0.612–0.729)
6–7 days/week	0.630 (0.588–0.676)	0.626 (0.584–0.672)	0.636 (0.593–0.681)
*p*-value	<0.001	<0.001	<0.001
MASLD			
Never	Ref.	Ref.	Ref.
< 1 day/week	0.893 (0.844–0.945)	0.914 (0.865–1.006)	0.901 (0.852–1.004)
1–2 days/week	0.796 (0.733–0.863)	0.798 (0.735–0.865)	0.803 (0.741–0.871)
3–5 days/week	0.588 (0.536–0.645)	0.576 (0.524–0.633)	0.580 (0.528–0.637)
6–7 days/week	0.545 (0.506–0.587)	0.541 (0.502–0.583)	0.554 (0.515–0.596)
*p*-value	<0.001	<0.001	<0.001
Advanced liver fibrosis^d^			
Never	Ref.	Ref.	Ref.
< 1 day/week	0.928 (0.817–1.055)	0.930 (0.818–1.057)	0.930 (0.818–1.057)
1–2 days/week	0.968 (0.814–1.150)	0.966 (0.813–1.149)	0.967 (0.813–1.149)
3–5 days/week	0.903 (0.754–1.083)	0.907 (0.757–1.087)	0.903 (0.754–1.083)
6–7 days/week	0.975 (0.846–1.124)	0.975 (0.846–1.124)	0.975 (0.845–1.124)
*p*-value	0.736	0.765	0.751
Advanced liver fibrosis^e^			
Never	Ref.	Ref.	Ref.
< 1 day/week	0.881 (0.772–1.005)	0.879 (0.771–1.003)	0.881 (0.773–1.005)
1–2 days/week	0.981 (0.824–1.168)	0.977 (0.821–1.164)	0.979 (0.822–1.166)
3–5 days/week	0.943 (0.787–1.130)	0.939 (0.784–1.126)	0.941 (0.785–1.127)
6–7 days/week	0.988 (0.855–1.140)	0.984 (0.852–1.136)	0.987 (0.855–1.140)
*p*-value	0.338	0.337	0.344

HR, hazard ratio; CI, confidential interval; Ref., reference; NAFLD, non-alcoholic fatty liver disease; MASLD, metabolic dysfunction-associated steatotic liver disease.

^a^Model I: Crude HR and 95% CI.

^b^Model II: adjusted for stratification by demographic factors.

^c^Model III: additionally adjusted for dietary preferences, and family medical history.

^d^Advanced liver fibrosis was evaluated using the NAFLD Fibrosis Score (NFS), with advanced liver fibrosis defined as NFS > − 1.455.

^e^Advanced liver fibrosis was evaluated using the Fibrosis-4 index (FIB-4), with advanced liver fibrosis defined as FIB-4 > 1.3.

**Figure 1:**
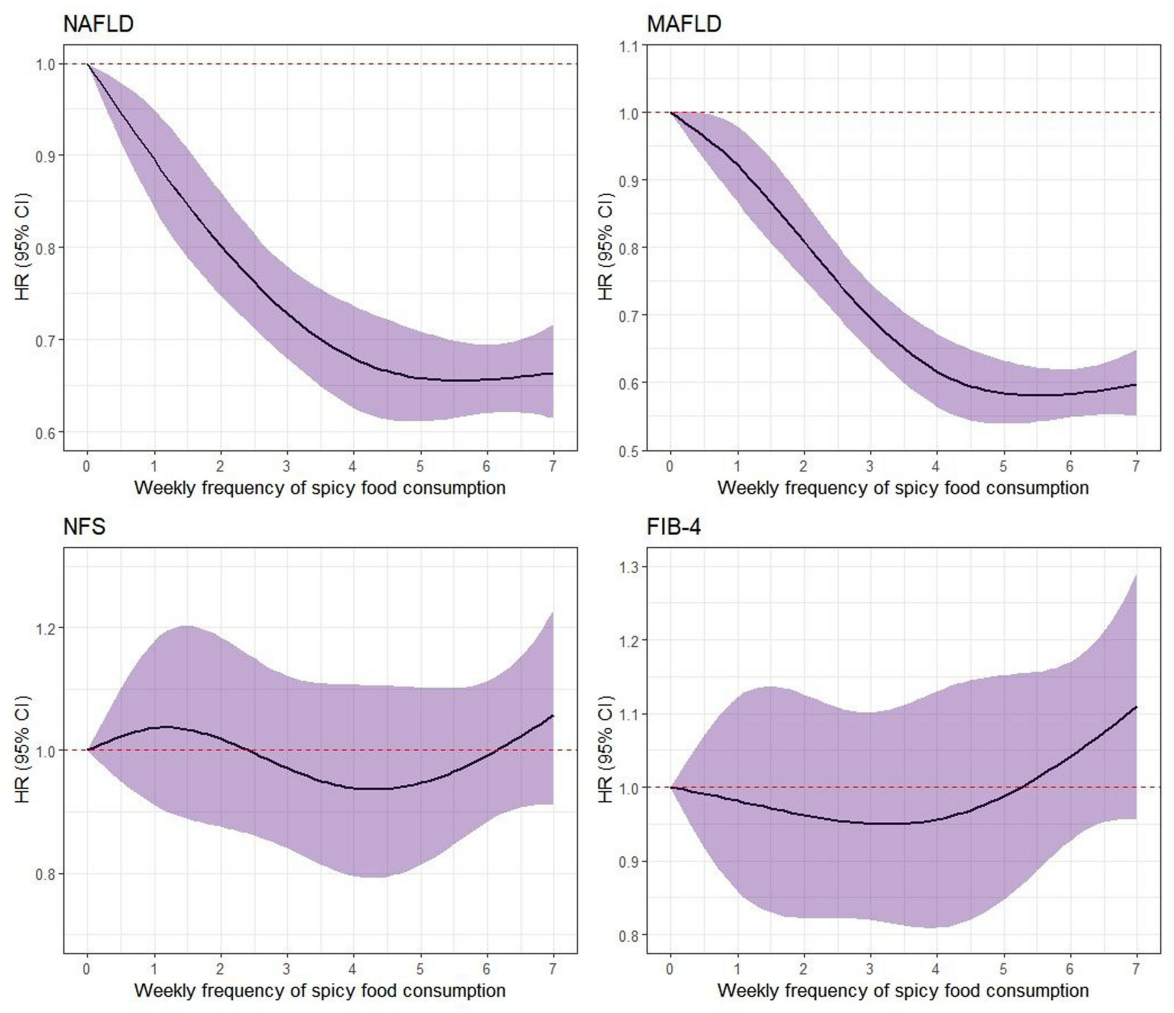
Nonlinear association between the frequency of weekly consumption of spicy foods and the risk of NAFLD/MASLD as well as liver fibrosis. The associations were evaluated using multi-variable Cox regression models with restricted cubic splines. NAFLD, non-alcoholic fatty liver disease; MASLD, metabolic dysfunction-associated steatotic liver disease; NFS, NAFLD Fibrosis Score; FIB-4, Fibrosis-4 index.

A significant non-linear relationship between the frequency of weekly consumption of spicy foods and the risk of NAFLD/MASLD (*p* < 0.001) was illustrated in Figure 1. However, this relationship was not observed in advanced liver fibrosis for either diagnostic standard.

### Subgroup and sensitivity analyses

The results of subgroup analyses stratified by age, gender, smoking status, and educational level, working intensity, household income, and marital status are presented in Table 3. Compared to the overall analysis, the consistent findings from subgroup analyses suggested that individuals who consumed spicy foods exhibited a lower risk of NAFLD/MASLD than those who did not engage in spicy food consumption. Furthermore, we did not observe significant changes in the subgroup analyses related to advanced liver fibrosis (Supplementary Table 3).

**Table 3:** Subgroup analysis of the risk associated with NAFLD/MASLD based on the frequency of weekly consumption of spicy foods.

Variables	Subgroups	NAFLD (HR [95% CI])^*^					MASLD (HR [95% CI])^*^				
		Never	< 1 day/week	1–2 days/week	3–5 days/week	6–7 days/week	Never	< 1 day/week	1–2 days /week	3–5 days/week	6–7 days/week
Age group	<45 y	Ref.	0.954 (0.883–1.029)	0.810 (0.724–0.906)	0.694 (0.614–0.783)	0.635 (0.576–0.699)	Ref.	0.894 (0.825–1.068)	0.793 (0.706–0.891)	0.605 (0.530–0.690)	0.549 (0.495–0.610)
	≥45 y	Ref.	0.933 (0.863–1.009)	0.813 (0.728–0.907)	0.644 (0.569–0.729)	0.636 (0.576–0.703)	Ref.	0.907 (0.839–1.082)	0.818 (0.732–0.914)	0.558 (0.489–0.637)	0.559 (0.504–0.621)
Sex	Female	Ref.	0.938 (0.868–1.013)	0.814 (0.728–0.910)	0.645 (0.571–0.729)	0.614 (0.557–0.678)	Ref.	0.895 (0.826–1.069)	0.848 (0.757–0.949)	0.590 (0.518–0.672)	0.557 (0.501–0.618)
	Male	Ref.	0.947 (0.877–1.023)	0.807 (0.722–0.901)	0.691 (0.611–0.782)	0.656 (0.595–0.723)	Ref.	0.906 (0.838–1.080)	0.768 (0.685–0.861)	0.573 (0.501–0.655)	0.552 (0.497–0.612)
Smoking status	Yes	Ref.	0.866 (0.742–1.009)	0.768 (0.612–0.964)	0.508 (0.392–0.657)	0.645 (0.534–0.778)	Ref.	0.893 (0.759–1.050)	0.866 (0.688–1.091)	0.586 (0.452–0.760)	0.644 (0.527–0.787)
	No	Ref.	0.955 (0.901–1.012)	0.818 (0.752–0.889)	0.694 (0.633–0.761)	0.633 (0.588–0.682)	Ref.	0.902 (0.849–0.997)	0.799 (0.733–0.870)	0.580 (0.525–0.641)	0.541 (0.500–0.586)
Educational level	Low	Ref.	0.952 (0.891–1.018)	0.815 (0.740–0.898)	0.683 (0.614–0.760)	0.632 (0.581–0.688)	Ref.	0.894 (0.834–1.057)	0.824 (0.747–0.909)	0.596 (0.531–0.669)	0.550 (0.503–0.602)
	High	Ref.	0.925 (0.841–1.017)	0.800 (0.699–0.916)	0.639 (0.551–0.742)	0.643 (0.571–0.726)	Ref.	0.916 (0.832–1.009)	0.773 (0.673–0.889)	0.554 (0.472–0.651)	0.563 (0.495–0.640)
Working intensity	Light	Ref.	0.939 (0.887–0.995)	0.817 (0.752–0.886)	0.678 (0.619–0.743)	0.634 (0.589–0.681)	Ref.	0.893 (0.842–0.948)	0.789 (0.725–0.859)	0.584 (0.530–0.645)	0.544 (0.504–0.588)
	Heavy	Ref.	0.974 (0.820–1.158)	0.743 (0.563–0.980)	0.588 (0.444–0.778)	0.654 (0.522–0.819)	Ref.	0.975 (0.816–1.166)	1.001 (0.774–1.293)	0.558 (0.415–0.750)	0.654 (0.519–0.826)
	High	Ref.	0.921 (0.826–1.027)	0.803 (0.687–0.938)	0.617 (0.515–0.738)	0.557 (0.482–0.643)	Ref.	0.904 (0.809–1.011)	0.809 (0.691–0.946)	0.585 (0.486–0.704)	0.550 (0.475–0.637)
Marriage status	Couples	Ref.	0.941 (0.887–0.998)	0.794 (0.729–0.865)	0.649 (0.591–0.713)	0.625 (0.580–0.673)	Ref.	0.890 (0.837–1.046)	0.794 (0.728–0.866)	0.552 (0.499–0.612)	0.550 (0.508–0.596)
	Singles	Ref.	0.957 (0.831–1.102)	0.915 (0.746–1.122)	0.782 (0.626–0.978)	0.700 (0.587–0.836)	Ref.	0.961 (0.832–1.110)	0.878 (0.711–1.085)	0.765 (0.607–0.963)	0.576 (0.475–0.699)

HR, hazard ratio; CI, confidential interval; Ref., reference; y, years old; NAFLD, non-alcoholic fatty liver disease; MASLD, metabolic dysfunction-associated steatotic liver disease.

^*^HR was adjusted for stratification by demographic factors, dietary preferences, and family medical history.

The robustness of the results was evaluated through sensitivity analyses conducted from three distinct perspectives, as outlined in Table 4. Firstly, excluding participants with missing data did not compromise the integrity of the primary findings. Secondly, consistency was observed between the dataset without missing values and the dataset utilizing multiple imputation techniques. Finally, after removing participants with a family medical history at baseline, it was evident that consumption of spicy foods remained associated with a lower risk of NAFLD/MASLD compared to those who never consumed spicy foods.

**Table 4:** Sensitivity analyses of adjusted hazard ratios for the risk of NAFLD/MASLD and liver fibrosis, based on the frequency of weekly consumption of spicy foods.

Health outcomes	HR [95% CI]^a^	HR [95% CI]^b^	HR [95% CI]^c^
NAFLD			
Never	Ref.	Ref.	Ref.
< 1 day/week	0.954(0.900–1.011)	0.957(0.892–1.027)	0.953(0.874–1.040)
1–2 days/week	0.820(0.756–0.890)	0.840(0.757–0.932)	0.779(0.688–0.881)
3–5 days/week	0.675(0.618–0.738)	0.667(0.595–0.748)	0.701(0.614–0.801)
6–7 days/week	0.643(0.598–0.691)	0.649(0.593–0.710)	0.631(0.567–0.702)
*p*-value	<0.001	<0.001	<0.001
MASLD			
Never	Ref.	Ref.	Ref.
< 1 day/week	0.888(0.837–1.043)	0.920(0.856–1.019)	0.880(0.805–1.061)
1–2 days/week	0.796(0.732–0.864)	0.826(0.742–0.919)	0.750(0.661–0.850)
3–5 days/week	0.573(0.521–0.631)	0.584(0.516–0.661)	0.545(0.471–0.631)
6–7 days/week	0.546(0.506–0.591)	0.561(0.509–0.618)	0.525(0.469–0.589)
*p*-value	<0.001	<0.001	<0.001
Advanced liver fibrosis^d^			
Never	Ref.	Ref.	Ref.
< 1 day/week	0.932(0.813–1.068)	0.914(0.773–1.081)	0.863(0.705–1.058)
1–2 days/week	0.972(0.812–1.162)	0.965(0.766–1.216)	0.954(0.733–1.242)
3–5 days/week	0.907(0.752–1.094)	0.950(0.751–1.201)	0.957(0.730–1.254)
6–7 days/week	0.979(0.843–1.137)	1.024(0.851–1.232)	0.880(0.704–1.101)
*p*-value	0.637	0.697	0.551
Advanced liver fibrosis^e^			
Never	Ref.	Ref.	Ref.
< 1 day/week	0.905(0.786–1.042)	0.997(0.841–1.181)	0.925(0.746–1.146)
1–2 days/week	1.007(0.840–1.208)	0.972(0.765–1.235)	0.939(0.707–1.248)
3–5 days/week	0.968(0.802–1.168)	0.931(0.729–1.189)	0.903(0.672–1.214)
6–7 days/week	1.015(0.871–1.182)	1.013(0.836–1.227)	1.028(0.817–1.294)
*p*-value	0.410	0.236	0.472

HR, hazard ratio; CI, confidential interval; Ref., reference; NAFLD, non-alcoholic fatty liver disease; MASLD, metabolic dysfunction-associated steatotic liver disease.

^a^The participants who had missing data were excluded from the analyses.

^b^The participants with missing data were included in the analysis without employing multiple imputation methods.

^c^The participants who reported a family medical history were excluded from the analyses.

^d^Advanced liver fibrosis was evaluated using the NAFLD Fibrosis Score (NFS), with advanced liver fibrosis defined as NFS > −1.455.

^e^Advanced liver fibrosis was evaluated using the Fibrosis-4 index (FIB-4), with advanced liver fibrosis defined as FIB-4 >1.3.

### Chili level preference and NAFLD/MASLD/advanced liver fibrosis

In further studies, we investigated the relationship between chili level preference and NAFLD/MASLD, as well as advanced liver fibrosis. As presented in Table 5, participants who never consumed or consumed spicy foods less than once per week had a 22.9% lower risk of NAFLD (HR = 0.771, 95%CI = 0.719–0.825) and a 24.6% lower risk of MASLD (HR = 0.754, 95%CI = 0.701–0.810) compared to those preferring mild pungency in their diet. Participants consuming spicy food with moderate pungency exhibited a 33.9% reduction in the risk of NAFLD (HR = 0.661, 95%CI = 0.570–0.761) and a remarkable 43.3% decrease in the risk of MASLD (HR = 0.567, 95%CI = 0.478–0.666). However, no significant association was observed between heavy pungency preference and either NAFLD or MASLD outcomes; furthermore, we did not find any significant correlation between chili level preference and advanced liver fibrosis.

**Table 5:** Adjusted hazard ratios for the risk of NAFLD/MASLD and liver fibrosis, stratified by the chili level preference.

Health outcomes	Model I (HR [95% CI])^a^	Model II (HR [95% CI])^b^	Model III (HR [95% CI])^c^
NAFLD			
Non-consuming	Ref.	Ref.	Ref.
Mild pungency	0.759 (0.708–0.812)	0.730 (0.679–0.784)	0.771 (0.719–0.825)
Moderate pungency	0.643 (0.553–0.742)	0.618 (0.528–0.718)	0.661 (0.570–0.761)
Heavy pungency	0.963 (0.853–1.086)	0.984 (0.874–1.107)	0.983 (0.873–1.106)
*p*-value	<0.001	<0.001	<0.001
MASLD			
Non-consuming	Ref.	Ref.	Ref.
Mild pungency	0.773 (0.720–0.829)	0.714 (0.661–0.770)	0.754 (0.701–0.810)
Moderate pungency	0.587 (0.498–0.687)	0.527 (0.439–0.626)	0.567 (0.478–0.666)
Heavy pungency	1.014 (0.898–1.145)	1.002 (0.886–1.132)	1.005 (0.889–1.135)
*p*-value	<0.001	<0.001	<0.001
Advanced liver fibrosis^d^			
Non-consuming	Ref.	Ref.	Ref.
Mild pungency	0.932 (0.817–1.061)	0.914 (0.799–1.043)	0.912 (0.797–1.041)
Moderate pungency	0.809 (0.602–1.062)	0.789 (0.583–1.043)	0.879 (0.672–1.132)
Heavy pungency	1.090 (0.875–1.372)	1.112 (0.897–1.395)	1.140 (0.925–1.422)
*p*-value	0.667	0.576	0.517
Advanced liver fibrosis^e^			
Non-consuming	Ref.	Ref.	Ref.
Mild pungency	0.930 (0.811–1.064)	0.899 (0.780–1.032)	0.907 (0.788–1.041)
Moderate pungency	0.859 (0.650–1.117)	0.838 (0.630–1.096)	0.813 (0.606–1.071)
Heavy pungency	1.079 (0.870–1.359)	1.081 (0.871–1.362)	1.060 (0.850–1.341)
*p*-value	0.833	0.747	0.431

HR, hazard ratio; CI, confidential interval; Ref., reference; NAFLD, non-alcoholic fatty liver disease; MASLD, metabolic dysfunction-associated steatotic liver disease.

^a^Model I: Crude HR and 95% CI.

^b^Model II: adjusted for stratification by demographic factors.

^c^Model III: additionally adjusted for dietary preferences, and family medical history.

^d^Advanced liver fibrosis was evaluated using the NAFLD Fibrosis Score (NFS), with advanced liver fibrosis defined as NFS > − 1.455.

^e^Advanced liver fibrosis was evaluated using the Fibrosis-4 index (FIB-4), with advanced liver fibrosis defined as FIB-4 > 1.3.

## Discussion

In our study, we investigated the association between weekly consumption of spicy food and the risk of NAFLD/MASLD, as well as advanced liver fibrosis, using a large cohort from the Chinese population. Our findings indicated that weekly consumption of spicy foods was inversely associated with the incidence of NAFLD/MASLD; however, no such association was observed for advanced liver fibrosis. Furthermore, we identified an inverse dose–response relationship between spicy food intake and the risk of developing incident NAFLD/MASLD.

Studies suggested that capsaicin may contribute to the improvement of NAFLD by reducing hepatic lipid accumulation, alleviating oxidative stress, and diminishing inflammation. These effects indicated that capsaicin had the potential to serve as a therapeutic agent for the management of NAFLD and its progression to more severe liver diseases (41). The definition of MASLD was fundamentally linked to the presence of indicator of cardiometabolic dysregulation. These indicators included BMI, WC, blood pressure, TG, HDL, and FBG. Therefore, managing cardiometabolic dysregulation may offer advantages in the prevention of MASLD, potentially improving overall health outcomes. In the context of obesity, several studies have demonstrated that capsaicinoids can enhance the effects of caloric restriction on weight loss. Supplementation with capsaicin has been shown to alleviate increases in hunger and decreases in feelings of satiety, as well as mitigate reductions in energy expenditure and fat oxidation typically associated with caloric restriction. These benefits may contribute to delaying the onset of resistance to fat loss during weight management efforts and assist in maintaining body weight following periods of obesity. Furthermore, evidence suggested that capsaicin may indirectly influence energy balance through its analgesic properties, which could improve sleep quality and support overall energy regulation (42, 43). Regarding blood pressure, existing studies indicate that the consumption of spicy foods may lead to a reduction in DBP and SBP, demonstrating an anti-hypertensive effect within the population-based cohort from the Sichuan Basin, China (44). A similar trend was observed in another large national cohort study conducted in China (45). In addition, a meta-analysis of seven trials involving 363 subjects indicated that capsinoids derived from fermented red pepper paste were significantly associated with a reduction in DBP. Specifically, the consumption of capsinoids (≤200 mg) and fermented red pepper paste (11.9 g) demonstrated a significant association with decreased DBP (24). Regarding TG and HDL, numerous animal studies have demonstrated that dietary capsaicin can reduce liver steatosis in obese mice subjected to a high-fat diet by inducing peroxisome proliferator-activated receptor *α* (PPAR-α) (46), lowering TG levels and reducing the expression of inflammatory adipocytokine genes (47). Recent studies have demonstrated that the supplementation of capsaicinoids lowered blood lipid levels and enhanced cholesterol metabolism (28). For FBG, cohort studies suggested that consumption of spicy foods may be associated with a reduced risk of development type 2 DM, particularly when consumed three to five days per week with mild pungency (20). Animal studies indicated that capsaicin reduced the proliferation and activation of autoreactive T cells in pancreatic lymph nodes, providing protection against type 1 DM in mice (48). In cases of type 2 DM, dietary capsaicin activates transient receptor potential vanilloid subfamily 1 (TRPV1), which enhanced glucose homeostasis while increasing plasma and ileal glucagon-like peptide-1 (GLP-1) levels (49). More specific research findings indicated that an intake of 5 mg of capsaicin was associated with improved blood sugar metabolism, which was roughly equivalent to the consumption of approximately 2–4 grams of chili pepper (50). Therefore, the consumption of spicy foods offered benefits for anti-obesity, anti-hypertension, anti-dyslipidemia, and anti-diabetes effects, potentially protecting against liver damage as well as the incidence of NAFLD/MASLD.

Although our study did not observe a benefit of spicy food consumption on liver fibrosis, previous *in vivo* and *in vitro* studies have demonstrated that the intake of spicy foods may contribute to the prevention of liver fibrosis. Three main mechanisms inhibiting advanced liver fibrosis have been identified in prior studies. Firstly, capsaicin targeted Notch signaling to inhibit M1 macrophage polarization, reducing tumor necrosis factor-*α* (TNF-α) secretion and weakening myofibroblast regeneration and hepatic stellate cells (HSCs) fibrosis formation (51). Secondly, capsaicin activated PPAR-*γ* to inhibit the transforming growth factor -β1 (TGF-β1)/Smad pathway, improving advanced liver fibrosis (52). Lastly, capsaicin reduced cell proliferation, activation, hydrogen peroxide production, and lowers tissue inhibitor of metalloproteinases-1 (TIMP-1) and TGF-1 levels (53). There were two established mouse models, namely bile duct ligation (BDL) and carbon tetrachloride (CCl4), that have demonstrated the inhibitory effect of dietary capsaicin on advanced liver fibrosis *in vivo*. This was evidenced by a reduction in fibrosis-related damage, decreased deposition of collagen and *α*-smooth muscle actin (αSMA)^+^ cells, as well as lowered expression levels of profibrogenic markers in isolated HSCs (54). Following the aforementioned three mechanisms, capsaicin effectively mitigated advanced liver fibrosis by inhibiting the proliferation of HSCs and promoting cellular apoptosis.

Among the capsaicinoids, capsaicin and dihydrocapsaicin exhibited the most pronounced burning sensation, as they account for 90% of the total pungency of pepper fruit (55). Therefore, the level of pungency in peppers was primarily determined by these compounds. He et al. reported that female participants who consumed spicy food exhibited a higher incidence risk of hypertension compared to those who did not consume such foods, particularly when consuming moderately pungent foods (45). Kenig et al. found that moderate consumption of chili pepper sauce—rather than high daily intake—resulted in decreased serum glucose, LDL cholesterol, and CRP levels in healthy individuals (56). Chen et al. indicated that regular consumption of spicy food may lower the risk of developing type 2 DM, especially at frequencies ranging from three to five days per week and with mild pungency (20). Interestingly, we observed that participants preferring weak to moderate levels of spiciness had a reduced risk of NAFLD/MASLD. Therefore, our findings were comprehensively supported by evidence from previous studies. Regarding mechanistic investigations, available data suggested that acute exposure to high levels of capsaicin can lower blood pressure by downregulating calcitonin gene-related peptide (CGRP), a vasodilatory substance released from sensory nerve terminals (57). Furthermore, additional analyses revealed that while moderate exposure to capsaicin did not alter plasma levels of CGRP, it was associated with increased plasma nitric oxide levels linked to improve blood pressure regulation. This finding suggested that the blood pressure-lowering effect attributed to capsaicin may be contingent upon its level of pungency (58).

To the best of our knowledge, this study is the first to explore the association between spicy food consumption and the risk of NAFLD/MASLD as well as liver fibrosis. This investigation was characterized by a large sample size, adjustments for key dietary factors, and a population-based cohort design. Nevertheless, several limitations should be acknowledged in our research. Firstly, spicy food consumption frequency was self-reported based on past experiences, which may introduce recall bias into the analyses. Additionally, the assessment of spiciness intensity was subjective and could lead to measurement errors. Secondly, while we accounted for major socioeconomic factors, lifestyle variables, and clinical parameters in our multivariable analyses, there may still exist residual confounding factors that are either unmeasured or unknown. Finally, it was important to note that these findings were derived from Chinese adults aged 25–60 years who reside in regions where spicy foods were consumed more frequently than in other countries. Therefore, caution should be exercised when extrapolating these results to other populations or geographical areas.

## Conclusion

In conclusion, the frequency of spicy food consumption may be inversely associated with the risk of NAFLD/MASLD, although this relationship did not extend to advanced liver fibrosis. Additionally, the protective effect of spicy foods on NAFLD/MASLD appeared to be linked to mild and moderate levels of spiciness. Overall, our findings provided evidence that incorporating spicy food into one’s diet could serve as a potential strategy for reducing the risk of NAFLD/MASLD.

## Data Availability

The raw data supporting the conclusions of this article will be made available by the authors, without undue reservation.

## Supplementary material

SUPPLEMENTARY FIGURE 1 Results of the distributions of imputed and observed values for missing data following multiple imputation. The observed data is depicted in gray, while the imputed data is represented in red. EAC, excessive alcohol consumption; WI, working intensity; EL, educational level; HI, household income; CLP, chili level preference; FMH, family medical history.
