# Supplementary material for: Association between spicy food consumption and the risk of non-alcoholic fatty liver disease/metabolic dysfunction-associated steatotic liver disease and liver fibrosis
Source: Front Nutr. 2025 Nov 26;12:1729349. doi: 10.3389/fnut.2025.1729349 (PMC12689368; doi:10.3389/fnut.2025.1729349)
Supplement: Supplementary file 2 [file Table_1.DOCX]

**Supplementary table 1.** The count and proportion of missing data across all variables.

| **Variables** | **Count of missing data** | **Proportion** |
| --- | --- | --- |
| Total number | 23666 | - |
| Age | 308 | 1.30% |
| Sex | 47 | 0.20% |
| Smoking status | 68 | 0.28% |
| Drinking status | 50 | 0.21% |
| Work intensity | 592 | 2.50% |
| Household income | 544 | 2.30% |
| Educational level | 47 | 0.20% |
| Married status | 686 | 2.90% |
| Vegetables | 615 | 2.60% |
| Fruits | 686 | 2.90% |
| Meat | 426 | 1.80% |
| Egg | 426 | 1.80% |
| BMI | 71 | 0.30% |
| WC | 379 | 1.60% |
| DBP | 47 | 0.20% |
| SBP | 75 | 0.32% |
| CHOL | 24 | 0.10% |
| TG | 544 | 2.30% |
| HDL | 544 | 2.30% |
| LDL | 237 | 1.00% |
| Alb | 260 | 1.10% |
| ALT | 379 | 1.60% |
| AST | 663 | 2.80% |
| PLT | 308 | 1.30% |
| HbA1 | 473 | 2.00% |
| FBG | 47 | 0.20% |
| TBil | 521 | 2.20% |
| DBil | 24 | 0.10% |
| Chili level preference | 592 | 2.50% |
| Family medical history | 639 | 2.70% |

Abbreviation: BMI, body mass index; WC, waist circumstance; DBP, diastolic blood pressure; SBP, systolic blood pressure; CHOL, total cholesterol; TG, Triglyceride; HDL, high density lipoprotein; LDL, low density lipoprotein; Alb, albumin; ALT, alanine aminotransferase; AST, aspartate aminotransferase; PLT, platelet; HbA1, glycosylated hemoglobin; FBG, fast blood glucose; TBil, total bilirubin; DBil, direct bilirubin.

**Supplementary table 2.** Logistic regression model results (p-values) for estimating missing data mechanisms.

| **Variables** | **NAFLD** | **MASLD** | **Advanced liver fibrosis ^a^** | **Advanced liver fibrosis ^b^** |
| --- | --- | --- | --- | --- |
| Age | 0.239 | 0.182 | 0.354 | 0.425 |
| Sex | 0.470 | 0.239 | 0.101 | 0.164 |
| Smoking status | 0.548 | 0.315 | 0.256 | 0.546 |
| Drinking status | 0.295 | 0.222 | 0.248 | 0.299 |
| Work intensity | 0.441 | 0.476 | 0.488 | 0.406 |
| Household income | 0.560 | 0.114 | 0.426 | 0.329 |
| Married status | 0.241 | 0.513 | 0.325 | 0.318 |
| Vegetables | 0.424 | 0.235 | 0.354 | 0.455 |
| Fruits | 0.260 | 0.256 | 0.214 | 0.435 |
| Meat | 0.376 | 0.133 | 0.237 | 0.446 |
| Egg | 0.450 | 0.373 | 0.211 | 0.453 |
| BMI | 0.328 | 0.121 | 0.167 | 0.581 |
| WC | 0.350 | 0.296 | 0.415 | 0.526 |
| DBP | 0.431 | 0.536 | 0.441 | 0.332 |
| SBP | 0.358 | 0.424 | 0.427 | 0.290 |
| CHOL | 0.209 | 0.454 | 0.232 | 0.270 |
| TG | 0.394 | 0.149 | 0.407 | 0.253 |
| HDL | 0.127 | 0.528 | 0.472 | 0.434 |
| LDL | 0.126 | 0.104 | 0.300 | 0.363 |
| Alb | 0.570 | 0.209 | 0.527 | 0.201 |
| ALT | 0.149 | 0.556 | 0.226 | 0.544 |
| AST | 0.418 | 0.106 | 0.357 | 0.409 |
| PLT | 0.174 | 0.337 | 0.266 | 0.567 |
| HbA1 | 0.450 | 0.464 | 0.250 | 0.293 |
| FBG | 0.558 | 0.123 | 0.134 | 0.314 |
| TBil | 0.201 | 0.281 | 0.162 | 0.525 |
| DBil | 0.320 | 0.183 | 0.148 | 0.403 |
| Chili level preference | 0.316 | 0.355 | 0.263 | 0.341 |
| Family medical history | 0.396 | 0.572 | 0.177 | 0.438 |

Abbreviation: NAFLD, non-alcoholic fatty liver disease; MASLD, metabolic dysfunction-associated steatotic liver disease.

^a^ Advanced liver fibrosis was evaluated using the NAFLD Fibrosis Score (NFS), with advanced liver fibrosis defined as NFS > -1.455.

^b^ Advanced liver fibrosis was evaluated using the Fibrosis-4 index (FIB-4), with advanced liver fibrosis defined as FIB-4 >1.3.

**Supplementary table 3.** Subgroup analysis of the risk associated with advanced liver fibrosis based on the frequency of spicy food consumption.

| **Variables** | **Subgroups** | **Advanced liver fibrosis (HR [95% CI[) * ^a^** | | | | |  | **Advanced liver fibrosis (HR[95% CI[) * ^b^** | | | | |
| --- | --- | --- | --- | --- | --- | --- | --- | --- | --- | --- | --- | --- |
|  |  | **Never** | **＜ 1 day /wk** | **1-2 days /wk** | **3-5 days /wk** | **6-7 days /wk** |  | **Never** | **＜ 1 day /wk** | **1-2 days /wk** | **3-5 days /wk** | **6-7 days /wk** |
| **Age group** | ＜45 y | Ref. | 0.963(0.806-1.152) | 1.000(0.783-1.276) | 0.812(0.621-1.062) | 0.987(0.810-1.203) |  | Ref. | 0.778(0.646-0.936) | 0.853(0.661-1.100) | 0.877(0.679-1.133) | 0.889(0.727-1.087) |
|  | ≥45 y | Ref. | 0.895(0.745-1.075) | 0.941(0.737-1.202) | 0.991(0.775-1.269) | 0.963(0.785-1.181) |  | Ref. | 1.002(0.831-1.210) | 1.127(0.885-1.436) | 1.023(0.791-1.321) | 1.107(0.901-1.362) |
| **Sex** | female | Ref. | 0.990(0.823-1.192) | 1.095(0.857-1.398) | 1.002(0.777-1.290) | 1.032(0.840-1.267) |  | Ref. | 0.921(0.760-1.116) | 1.037(0.804-1.336) | 1.033(0.801-1.333) | 1.057(0.859-1.302) |
|  | male | Ref. | 0.874(0.732-1.043) | 0.861(0.675-1.099) | 0.817(0.63-1.059) | 0.927(0.762-1.129) |  | Ref. | 0.845(0.705-1.012) | 0.931(0.732-1.183) | 0.860(0.664-1.113) | 0.925(0.758-1.129) |
| **Smoking status** | yes | Ref. | 0.840(0.577-1.223) | 1.016(0.616-1.675) | 0.754(0.440-1.290) | 0.819(0.536-1.250) |  | Ref. | 0.646(0.442-0.945) | 0.940(0.579-1.525) | 0.944(0.589-1.511) | 0.913(0.617-1.350) |
|  | no | Ref. | 0.939(0.820-1.076) | 0.964(0.802-1.159) | 0.926(0.764-1.123) | 0.997(0.857-1.159) |  | Ref. | 0.919(0.799-1.058) | 0.989(0.820-1.193) | 0.941(0.773-1.144) | 1.000(0.856-1.167) |
| **Educational level** | low | Ref. | 0.937(0.800-1.097) | 0.939(0.755-1.167) | 0.978(0.785-1.219) | 1.045(0.880-1.241) |  | Ref. | 0.914(0.777-1.074) | 1.026(0.828-1.271) | 0.990(0.792-1.237) | 1.009(0.846-1.204) |
|  | high | Ref. | 0.914(0.735-1.136) | 1.021(0.768-1.356) | 0.768(0.559-1.056) | 0.840(0.652-1.082) |  | Ref. | 0.819(0.653-1.028) | 0.898(0.664-1.214) | 0.861(0.631-1.174) | 0.948(0.740-1.216) |
| **Working intensity** | light | Ref. | 0.946(0.827-1.083) | 0.934(0.778-1.122) | 0.945(0.782-1.141) | 0.976(0.840-1.133) |  | Ref. | 0.896(0.781-1.028) | 0.935(0.777-1.125) | 0.962(0.796-1.162) | 0.95(0.816-1.106) |
|  | heavy | Ref. | 0.789(0.521-1.194) | 1.297(0.774-2.173) | 0.589(0.310-1.118) | 0.953(0.604-1.504) |  | Ref. | 0.751(0.478-1.178) | 1.529(0.899-2.600) | 0.789(0.426-1.464) | 1.413(0.903-2.211) |
| **Household income** | low | Ref. | 0.904(0.780-1.047) | 0.995(0.817-1.211) | 0.894(0.726-1.100) | 0.964(0.819-1.135) |  | Ref. | 0.871(0.747-1.016) | 1.009(0.825-1.236) | 1.011(0.823-1.242) | 1.001(0.847-1.183) |
|  | high | Ref. | 1.005(0.776-1.301) | 0.885(0.617-1.270) | 0.903(0.623-1.309) | 1.005(0.753-1.342) |  | Ref. | 0.907(0.704-1.170) | 0.905(0.641-1.279) | 0.762(0.518-1.121) | 0.952(0.717-1.263) |
| **Marriage status** | couples | Ref. | 0.940(0.817-1.080) | 0.997(0.828-1.200) | 0.901(0.740-1.096) | 0.964(0.826-1.126) |  | Ref. | 0.859(0.746-0.989) | 0.951(0.788-1.146) | 0.905(0.746-1.099) | 0.952(0.815-1.111) |
|  | singles | Ref. | 0.870(0.629-1.203) | 0.805(0.498-1.301) | 0.923(0.576-1.480) | 1.042(0.732-1.482) |  | Ref. | 1.032(0.718-1.485) | 1.190(0.732-1.934) | 1.193(0.724-1.968) | 1.234(0.833-1.826) |

Abbreviation: HR, hazard ratio; CI, confidential interval; Ref., reference; y, years old; NAFLD, non-alcoholic fatty liver disease; MASLD, metabolic dysfunction-associated steatotic liver disease.

* HR was adjusted for stratification by demographic factors, dietary preferences, and family medical history.

^a^ Advanced liver fibrosis was evaluated using the NAFLD Fibrosis Score (NFS), with advanced liver fibrosis defined as NFS > -1.455.

^b^ Advanced liver fibrosis was evaluated using the Fibrosis-4 index (FIB-4), with advanced liver fibrosis defined as FIB-4 >1.3.
